# Chronic inflammation in psoriasis promotes visceral adiposity associated with noncalcified coronary burden over time

**DOI:** 10.1172/jci.insight.142534

**Published:** 2020-11-19

**Authors:** Aparna Sajja, Khaled M. Abdelrahman, Aarthi S. Reddy, Amit K. Dey, Domingo E. Uceda, Sundus S. Lateef, Alexander V. Sorokin, Heather L. Teague, Jonathan Chung, Joshua Rivers, Aditya A. Joshi, Youssef A. Elnabawi, Aditya Goyal, Justin A. Rodante, Andrew Keel, Julie E. Alvarez, Benjamin Lockshin, Ronald Prussick, Evan Siegel, Martin P. Playford, Marcus Y. Chen, David A. Bluemke, Joel M. Gelfand, Nehal N. Mehta

**Affiliations:** 1 Johns Hopkins Hospital, Baltimore, Maryland, USA.; 2 National Heart, Lung, and Blood Institute, Bethesda, Maryland, USA.; 3 Jacobi Medical Center, New York, New York, USA.; 4 DermAssociates, Silver Spring, Maryland, USA.; 5 Washington Dermatology Center, Rockville, Maryland, USA; George Washington University, Washington, DC, USA.; 6 Arthritis and Rheumatism Associates, Rockville, Maryland, USA.; 7 University of Wisconsin School of Medicine and Public Health, Madison, Wisconsin, USA.; 8 University of Pennsylvania, Philadelphia, Pennsylvania, USA.

**Keywords:** Cardiology, Inflammation, Adipose tissue, Atherosclerosis, Obesity

## Abstract

**BACKGROUND:**

Psoriasis is a chronic inflammatory skin disease associated with increased obesity, noncalcified coronary artery burden (NCB), and incident myocardial infarction. Here, we sought to assess the relationship among inflammation, visceral adipose tissue (VAT), and NCB. Furthermore, we evaluated whether improvement in VAT would be associated with reduction in NCB over time in psoriasis.

**METHODS:**

Consecutive psoriasis patients underwent coronary CT angiography to quantify NCB and abdominal CT to calculate VAT at baseline (*n* = 237), 1 year (*n* = 176), and 4 years (*n* = 50).

**RESULTS:**

Patients with high levels of high-sensitivity C-reactive protein (hs-CRP) had significantly greater visceral adiposity (17,952.9 ± 849.2 cc^3^ vs. 13370.7 ± 806.8 cc^3^, *P* < 0.001) and noncalcified coronary burden (1.26 ± 0.03 vs. 1.07 ± 0.02 mm^2^) than those with low levels of hs-CRP. Those with higher levels of VAT had more systemic inflammation (hs-CRP, median [IQR], 2.5 mg/L [1.0–5.3 mg/L] vs. 1.2 mg/L [0.6–2.9 mg/L]), with approximately 50% higher NCB (1.42 ± 0.6 mm^2^ vs. 0.91 ± 0.2 mm^2^, *P* < 0.001). VAT associated with NCB in fully adjusted models (β = 0.47, *P* < 0.001). At 1-year follow-up, patients who had worsening hs-CRP had an increase in VAT (14,748.7 ± 878.1 cc^3^ to 15,158.7 ± 881.5 cc^3^; *P* = 0.03), whereas those who had improved hs-CRP improved their VAT (16,876.1 ± 915.2 cc^3^ to 16310.4 ± 889.6 cc^3^; *P* = 0.04). At 1 year, there was 10.3% reduction in NCB in those who had decreased VAT (β = 0.26, *P* < 0.0001), which persisted in a subset of patients at 4 years (β = 0.39, *P* = 0.003).

**CONCLUSIONS:**

Inflammation drives development of VAT, increased cardiometabolic risk, and NCB in psoriasis. Reduction of inflammation associated with reduction in VAT and associated with longitudinal improvement in NCB. These findings demonstrate the important role of inflammation in the development of VAT in humans and its effect on early atherogenesis.

**TRIAL REGISTRATION:**

ClinicalTrials.gov NCT01778569.

**FUNDING:**

This study was supported by the National Heart, Lung, and Blood Institute Intramural Research Program (HL006193-05), the NIH Medical Research Scholars Program, a public-private partnership supported jointly by the NIH and contributions to the Foundation for the NIH from the Doris Duke Charitable Foundation (no. 2014194), the American Association for Dental Research, the Colgate-Palmolive Company, Genentech, and Elsevier as well as private donors.

## Abstract 2

Inflammation is associated with development of visceral adiposity and coronary artery disease in humans.

## Graphical abstract

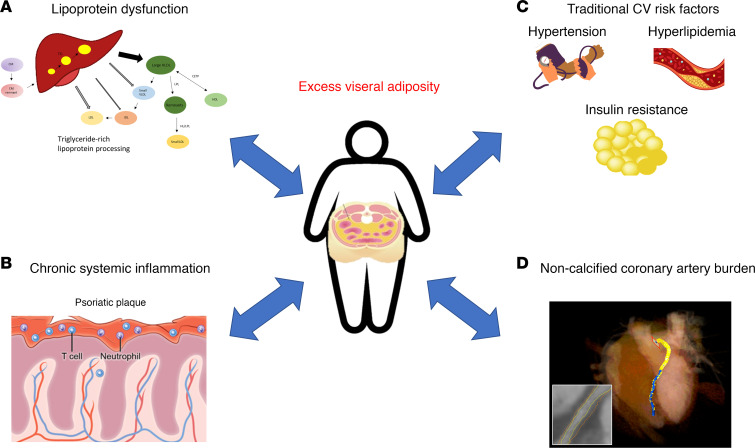

## Introduction

Inflammation plays a critical role in the development of cardiovascular disease (1). Psoriasis, a chronic inflammatory skin disease, is associated with high systemic and vascular inflammation, increased traditional cardiovascular (CV) risk factors, and accelerated coronary artery disease as well as myocardial infarction (2–5). Patients with psoriasis also tend to be overweight or obese and have increased risk of developing inflammatory atherogenesis (6–10). Given that coronary artery disease is a multifactorial disease, psoriasis serves as a useful model to understand the interplay of obesity, chronic inflammation, and dyslipidemia, all of which share pathophysiological pathways related to accelerated atherosclerosis (11).

Cardiometabolic risk has been difficult to characterize comprehensively in CV disease assessment (12, 13). With its rising prevalence, obesity has emerged as a driver of residual inflammatory risk (14–16). The regional distribution of adiposity, namely deep abdominal fat, referred to as visceral adipose tissue (VAT), has been shown to be associated with vascular inflammation and CV events independent of prevalent obesity, as assessed by BMI or subcutaneous adipose tissue (SAT) (17–19). From early endothelial cell dysfunction to coronary plaque rupture, inflammation has been shown to drive atherosclerosis (20, 21). VAT has been shown to be metabolically active, as it gives rise to inflammatory and proatherogenic factors and pathological adipokines, while increasing the rates of infiltrating macrophages and lymphocytes, compared with those in SAT, and, thus, better captures adipose dysfunction (22, 23). This is relevant because visceral adiposity has been associated with increased inflammation and subclinical vascular disease, including coronary artery disease, by coronary CT angiography (CCTA) (6, 24, 25). CCTA is a noninvasive imaging modality with strong prognostic utility, which allows for characterization of coronary artery characteristics, including noncalcified coronary artery burden (NCB), a strong predictor of CV events, over time (26, 27). Moreover, the accelerated CV risk seen in psoriasis is not adequately captured by traditional risk assessment and attributable to increased burden of subclinical coronary artery disease, with increased NCB, as measured by CCTA (5).

The interplay among inflammation, VAT, and coronary artery disease has not been previously studied in humans to our knowledge, particularly in a high-risk inflammatory disease state. Understanding these relationships among inflammation, VAT, and early coronary artery disease may permit the development of strategies targeting VAT to reduce cardiometabolic and CV disease development. Therefore, we aimed to test the hypotheses that (a) inflammation is associated with volume of VAT in psoriasis; (b) the volume of VAT is associated with CV risk factors, markers of inflammation, and early coronary artery disease, as assessed by NCB on CCTA; (c) over 1 year, persistent inflammation would be associated with development of more VAT; and (d) a reduction in the volume of VAT at 1 year and 4 years would associate with improvement in NCB.

## Results

### Characteristics of entire study group.

Chronic inflammation has been shown to play a crucial role in the development of obesity-related insulin resistance and infiltration of inflammatory cells into adipose tissue (28, 29). Therefore, we first stratified patients with psoriasis by systemic inflammation using median hs-CRP levels (hs-CRP >1.8 mg/L) (Table 1). Overall, the entire psoriasis sample (*n* = 237) was middle aged (age, 50.4 ± 12.8 years), predominantly male (60%), and had mild-to-moderate skin disease severity at baseline (Psoriasis Area Severity Index [PASI] score, median [IQR], 5.9 [3–10.4]). Participants had a low CV risk by Framingham 10-year risk score (median [IQR], 2 [1–6]), despite being overweight to obese (BMI, 28.6 kg/m^2^ [25–32.9 kg/m^2^]), and nearly half had a history of dyslipidemia (*n* = 96, 41%). Approximately one-third of participants were on statin therapy (*n* = 73, 31%) and one-third were on biologic therapy for their psoriasis (*n* = 81, 34%). In those with higher systemic inflammation, there was greater visceral adiposity (17,952.9 ± 849.2 cc^3^ vs. 13,370.7 ± 806.8 cc^3^, *P* < 0.001), BMI (median [IQR], 30.9 kg/m^2^ [27.7–36.2 kg/m^2^] vs. 29.5 kg/m^2^ [27.1–29.5 kg/m^2^]; *P* < 0.001), insulin resistance (Homeostatic Model Assessment of Insulin Resistance [HOMA-IR], median [IQR], 3.4 [2.1–6.1] vs. 2.2 [1.5–3.6], *P* < 0.001), and noncalcified coronary burden (1.26 ± 0.03 mm^2^ vs. 1.07 ± 0.02 mm^2^) than those with less inflammation (hs-CRP <1.8 mg/L) (Table 1).

### Characterization by extent of visceral adiposity.

**Figure 1:**
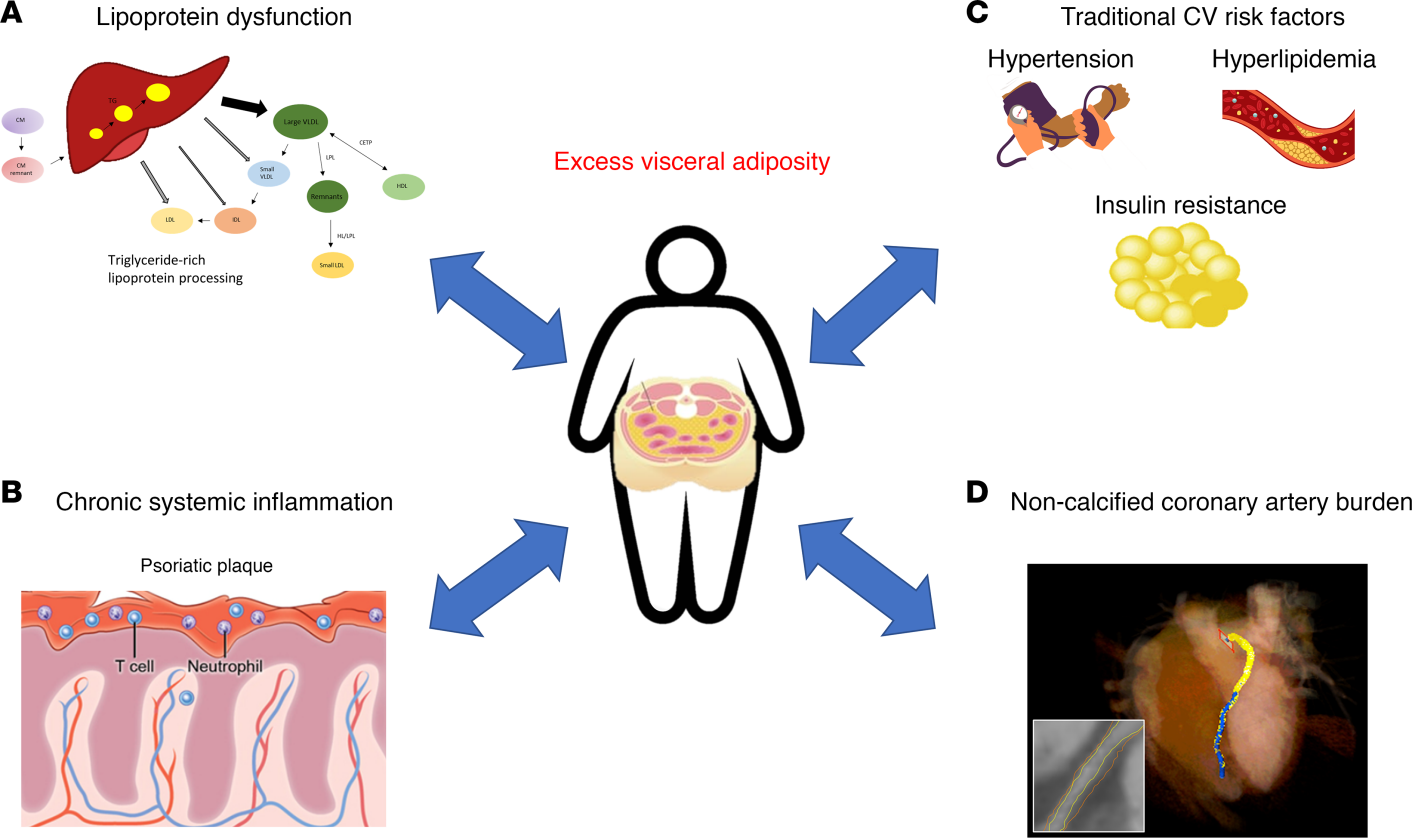
Systemic effects of visceral adiposity in psoriasis. (**A**) Psoriasis is associated with increased lipoprotein dysfunction, such as increased production of triglyceride-rich large VLDL particles. (**B**) High visceral adiposity is associated with greater systemic inflammation. (**C**) Furthermore, psoriasis is associated with traditional risk factors for atherosclerotic vascular disease. (**D**) Visceral adiposity increases cardiometabolic risk through associations with traditional risk factors, markers of lipid dysfunction, and systemic inflammation — contributing overall to increased noncalcified coronary artery burden, a marker of early coronary artery disease. CETP, cholesterylester transfer protein; CM, chylomicron; HDL, high density lipoprotein; HL, hepatic lipase; IDL, intermediate-density lipoprotein; LDL, low-density lipoprotein; LPL, lipoprotein lipase; TG, triglyceride; VLDL, very low-density lipoprotein**.**

**Figure 2:**
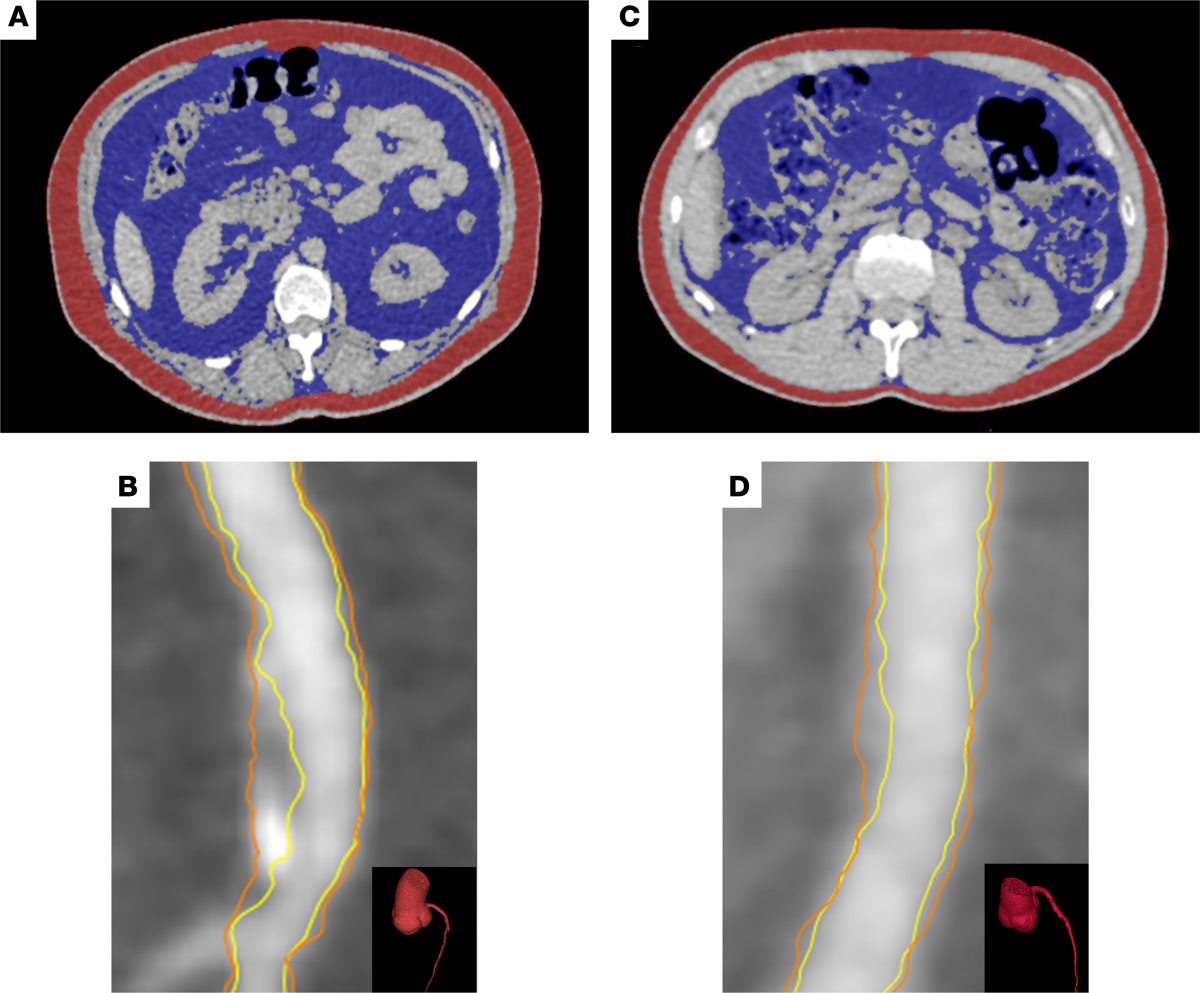
Visceral adiposity and noncalcified coronary artery burden in psoriasis. (**A**) Axial abdominal CT of a patient with psoriasis with high VAT volume (VAT, 32,767 cc^3^) for quantification of visceral (blue) and subcutaneous (red) adiposity. (**B**) CCTA showing the proximal left anterior descending artery (LAD) of the same patient, with the lumen (yellow) and the outer walls (orange) (LAD noncalcified coronary artery burden, 1.04 mm^2^) shown. (**C**) Axial abdominal CT of an age- and sex-matched patient with psoriasis with low VAT (VAT, 13,900 cc^3^) for quantification of visceral (blue) and subcutaneous (red) adiposity. (**D**) CCTA showing the LAD of the same patient, with the lumen (yellow) and the outer wall (orange) (LAD noncalcified coronary artery burden: 0.84 mm^2^) shown.

Table 2 reports patient characteristics that were stratified by high levels compared with low levels of VAT. Patients with psoriasis with high levels of VAT were older, male, and obese compared with patients with low levels of VAT. They also had an increased prevalence of traditional risk factors; more insulin resistance, as shown by HOMA-IR; and elevated systemic inflammation, as shown by GlycA and high-sensitivity C-reactive protein (hs-CRP) (*P* < 0.05). LDL particle, VLDL particle, and medium and large VLDL particle numbers were higher despite similar total cholesterol and LDL-cholesterol levels (*P* < 0.05). Figure 1 depicts the systemic effects of visceral adiposity in psoriasis. Patients with psoriasis with high levels of VAT had a greater NCB and coronary plaque prevalence compared with patients with low levels of VAT (*P* < 0.05) (Table 2 and Figure 2).

**Figure 3:**
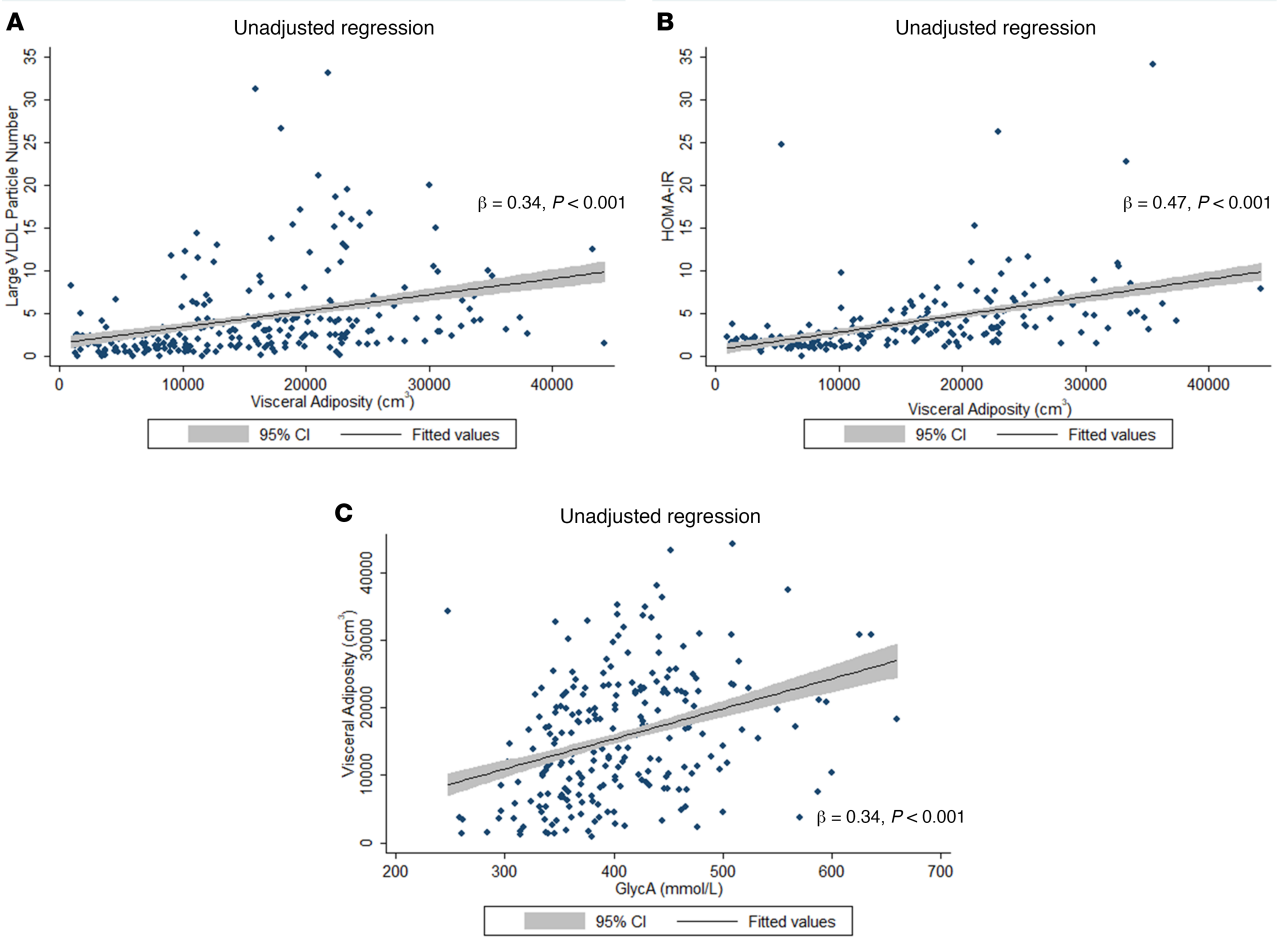
Relationship of visceral adiposity with lipid, metabolic, and inflammatory parameters. Fitted regression plot with data scatter showing the relationship of visceral adiposity with (**A**) lipid (large VLDL particle number), (**B**) metabolic (homeostatic model assessment of insulin resistance [HOMA-IR]), and (**C**) inflammatory (GlycA) parameters in psoriasis. The shaded regions represent the lower and upper 95% CIs for the fitted regression plot.

VAT significantly correlated with important inflammatory, lipid, and metabolic parameters in psoriasis (all *P* < 0.05; Figure 3 and Supplemental Table 1; supplemental material available online with this article; https://doi.org/10.1172/jci.insight.142534DS1). VAT correlated with traditional risk factors, such as hypertension, BMI, and Framingham 10-year risk score (all *P* < 0.05). Moreover, VAT associated with insulin resistance and markers of inflammation, such as psoriasis severity, hs-CRP, and GlycA (all *P* < 0.05). Finally, VAT associated with NMR lipid markers of atherogenic dyslipidemia, including LDL particle number, small LDL particle number, VLDL particle number, and medium VLDL particle and large VLDL particle number (all *P* < 0.05).

### Association between inflammation and development of visceral adiposity over time.

At 1-year follow-up, patients who had worsening hs-CRP (1.2 mg/L [0.7–2.7 mg/L] to 2.1 mg/L [0.9–3.8 mg/L]; *P* < 0.001) had an increase in VAT (14,748.7 ± 878.1 cc^3^ to 15,158.7 ± 881.5 cc^3^; *P* = 0.03), whereas those who had improved hs-CRP (3 mg/L [1.2–5.5 mg/L] to 1.3 mg/L [0.6–3.3 mg/L]; *P* < 0.001) had improved VAT (16,876.1 ± 915.2 cc^3^ to 16,310.4 ± 889.6 cc^3^; *P* = 0.04). In adjusted analysis, change in VAT associated with change in hs-CRP at 1 year (β = 0.19, *P* = 0.007). When adjusted for change in BMI at 1 year, this relationship remained robust (β = 0.22, *P* < 0.0001).

### Change in visceral adiposity over time and associations with noncalcified coronary burden over time.

**Figure 4:**
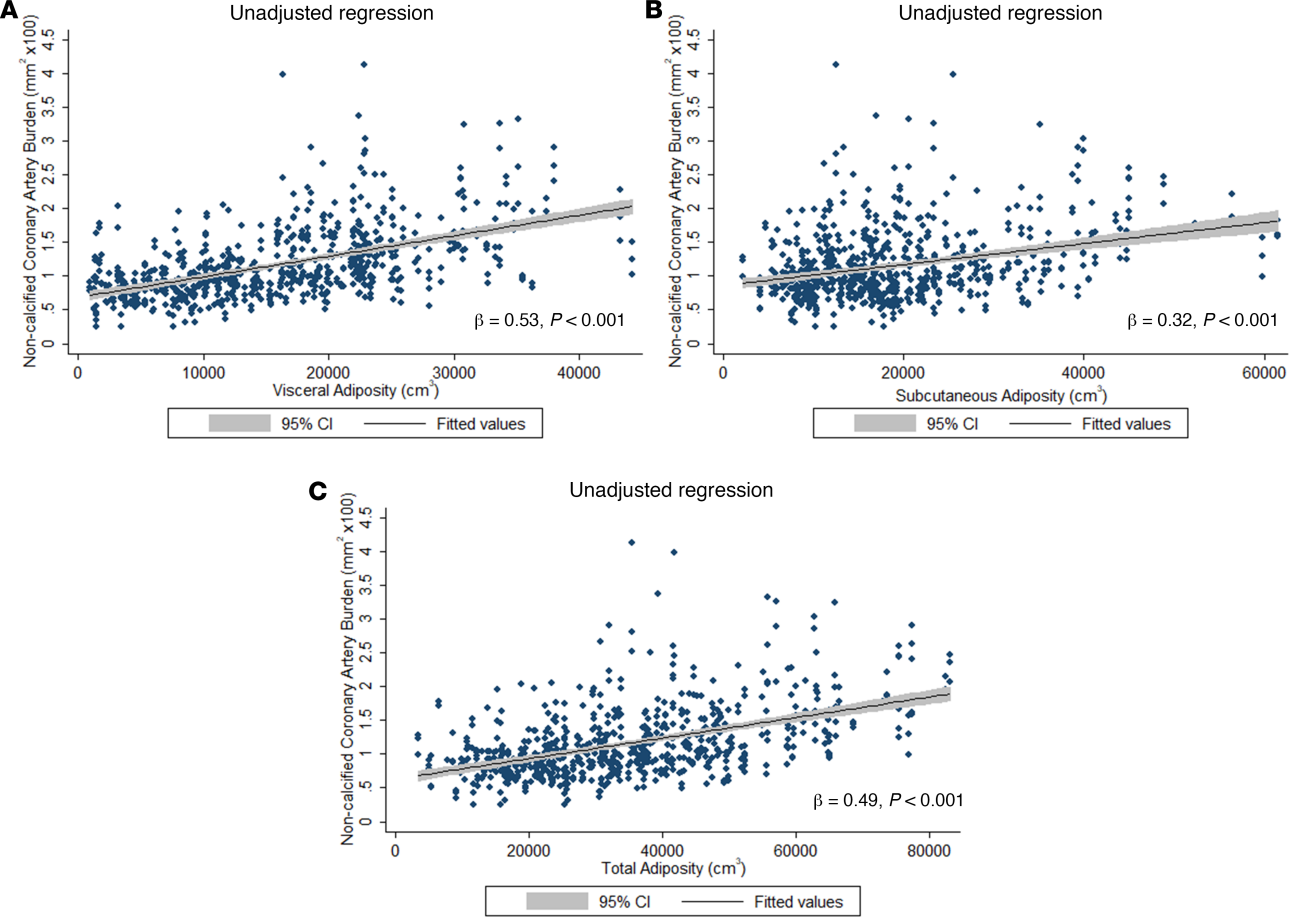
Relationship of adiposity with noncalcified coronary plaque burden in psoriasis. Fitted regression plot showing the relationship of (**A**) visceral, (**B**) subcutaneous, and (**C**) total adiposity with noncalcified coronary plaque burden. The shaded regions represent the lower and upper 95% CIs for the fitted regression plot.

VAT and NCB were associated in unadjusted models (β = 0.53 *P* < 0.001; Figure 4) and also after adjusting for Framingham risk score, hs-CRP, statin therapy, biologic therapy, and SAT by CT (β = 0.47; *P* < 0.001). Table 3 stratifies patients by improvement or worsening of VAT at 1-year follow-up. In patients who increased their visceral adiposity at 1 year, there was an increase in BMI, SAT, fasting blood glucose, and large VLDL particle number. This group also had a 9.9% increase in NCB (1.22 ± 0.60 mm^2^ as compared with 1.11 ± 0.48 mm^2^, *P* < 0.001). Conversely, patients who had improved visceral adiposity had decreases in BMI, SAT, LDL-cholesterol, and LDL particle number. This group also demonstrated improvement in psoriasis skin disease severity, hs-CRP, and GlycA, with a 10.3% decrease in noncalcified coronary burden (1.26 ± 0.57 mm^2^ vs. 1.13 ± 0.52 mm^2^, *P* < 0.001). Change in VAT directly associated with change in NCB at 1 year, independent of traditional CV risk factors, change in hs-CRP, and change in SAT, lipid, and biologic therapy at baseline and 1 year (β = 0.26, *P* < 0.0001). Finally, to understand whether these 1-year changes were durable at 4 years, we followed a consecutive sample of the first 50 patients with psoriasis in our cohort for 4 years (Supplemental Tables 2 and 3). The relationship between change in VAT and change in NCB persisted at 4 years, even after adjustment of traditional CV risk factors, change in hs-CRP, and change in SAT, lipid, and biologic therapy at baseline and 4 years (β = 0.39, *P* = 0.003).

## Discussion

Using a longitudinal cohort study design, we demonstrate that (a) inflammation is associated with volume of VAT in psoriasis; (b) patients with psoriasis with higher levels of visceral adiposity had higher cardiometabolic risk, including markers of inflammation, dyslipidemia, and NCB compared with those with low levels of VAT; (c) patients with psoriasis with worsening hs-CRP over time had increased visceral adiposity, while those who had improved hs-CRP over time had a reduction in VAT; and (d) the volume of VAT strongly associated with early coronary artery disease burden by NCB and at 1-year and 4-year follow-ups. Improvement in volume of VAT associated with improvement in NCB in fully adjusted models. These findings suggest the important role of inflammation in the development of visceral adiposity in humans and its effect on early atherogenesis.

Obesity is increasingly recognized as a driver of atherosclerotic CV disease and is highly prevalent in chronic inflammatory disease states such as psoriasis (30). Psoriasis is associated with cardiometabolic dysfunction, including atherogenic dyslipidemia, impaired lipoprotein function, and insulin resistance (31). Although BMI is a clinical marker of obesity at the population level, studies have shown that obesity is a heterogeneous condition with varying CV and metabolic manifestations (17, 18). Chronic inflammation has been shown to change adipose distribution and shift adiposity to metabolically active VAT (32, 33). Our data support that inflammation drives the increase in volume of VAT, which in turn may drive adipose dysfunction, leading to more inflammation and a dangerous cycle. Moreover, change in VAT associated with change in hs-CRP, beyond change in BMI at 1 year in our analyses, suggesting that VAT may inform cardiometabolic risk beyond BMI. We have previously shown that VAT is associated with vascular inflammation in psoriasis (6). Here, we demonstrate that VAT relates to this increased cardiometabolic risk in psoriasis through associations with traditional risk factors (including BMI), and markers of systemic inflammation, as assessed by hs-CRP, lipid dysfunction, and insulin resistance (Supplemental Table 1).

Chronic inflammation has been shown to play a critical role in development of obesity-related insulin resistance and visceral adiposity in preclinical and clinical models (28, 34–36). CRP levels are increased in individuals with excess VAT (37). Moreover, VAT is an immunogenic depot, promoting secretion of proinflammatory factors and activating macrophages. In psoriasis, this expanded visceral adiposity depot and proinflammatory state may lead to a relentless cycle, which in turn drives other pathways that impact development of atherosclerotic disease. Inflammatory VAT dysregulation may alter dysfunctional immune cell and adipokine secretion, exacerbating pathological endothelial changes and contributing to mechanisms of adiposity-related inflammatory atherogenesis (38).

We followed patients for 1 year and then a smaller subset of consecutive patients at 4 years. At 1 year, in those who had reduced VAT there was a reduction in NCB, while those who had increased VAT had significantly increased NCB. Both groups improved their PASI scores and had comparable increases in use of biologic treatment. Furthermore, those who had a favorable change in VAT at 4 years also had a reduction in NCB independent of traditional risk factors, whereas those who had increased VAT had progression of their NCB. These findings suggest a durable, long-term effect that VAT imparts on early vascular disease progression.

Our observation of improvement in VAT associating with improvement in NCB at 1- and 4-year follow-up provides important evidence to further study the biological mechanisms involved in inflammatory-modulated adipose dysfunction in humans. Future studies should focus on adipose biopsies before and after antiinflammatory therapy as well as aggressive lifestyle CV risk reduction measures, including intensive weight, diet, exercise, and lipid management. In fact, recently, exercise was shown to enhance antiinflammatory phenotype through accumulation of alternatively activated M2 macrophages in response to endurance exercise training in aging mice, supporting the importance of exercise in altering inflammatory pathways to modulate visceral adiposity (39).

### Study limitations.

Our study has notable limitations. It is observational in nature and therefore there is subjected to unmeasured confounders that may not be accounted for in our association analyses between VAT and NCB. Additionally, we have not reported CV events and instead used noncalcified burden as a surrogate of early CV disease risk. Finally, our study has been performed in a chronic inflammatory disease, psoriasis, and therefore may not be generalizable, although our study provides strong links between visceral adiposity and subclinical atherosclerosis.

### Conclusions.

In conclusion, inflammation drives VAT development, increased cardiometabolic risk, and noncalcified coronary burden in psoriasis, suggesting the role of inflammation-associated adipose dysfunction in humans over time and its relationship to early CV disease in psoriasis. Furthermore, improvement of visceral adiposity associated with an improvement in NCB in patients with psoriasis at 1-year and 4-year follow-ups, suggesting an important role in reducing inflammation to curb downstream cardiometabolic disease risk.

## Methods

### Study design and population.

**Figure 5:**
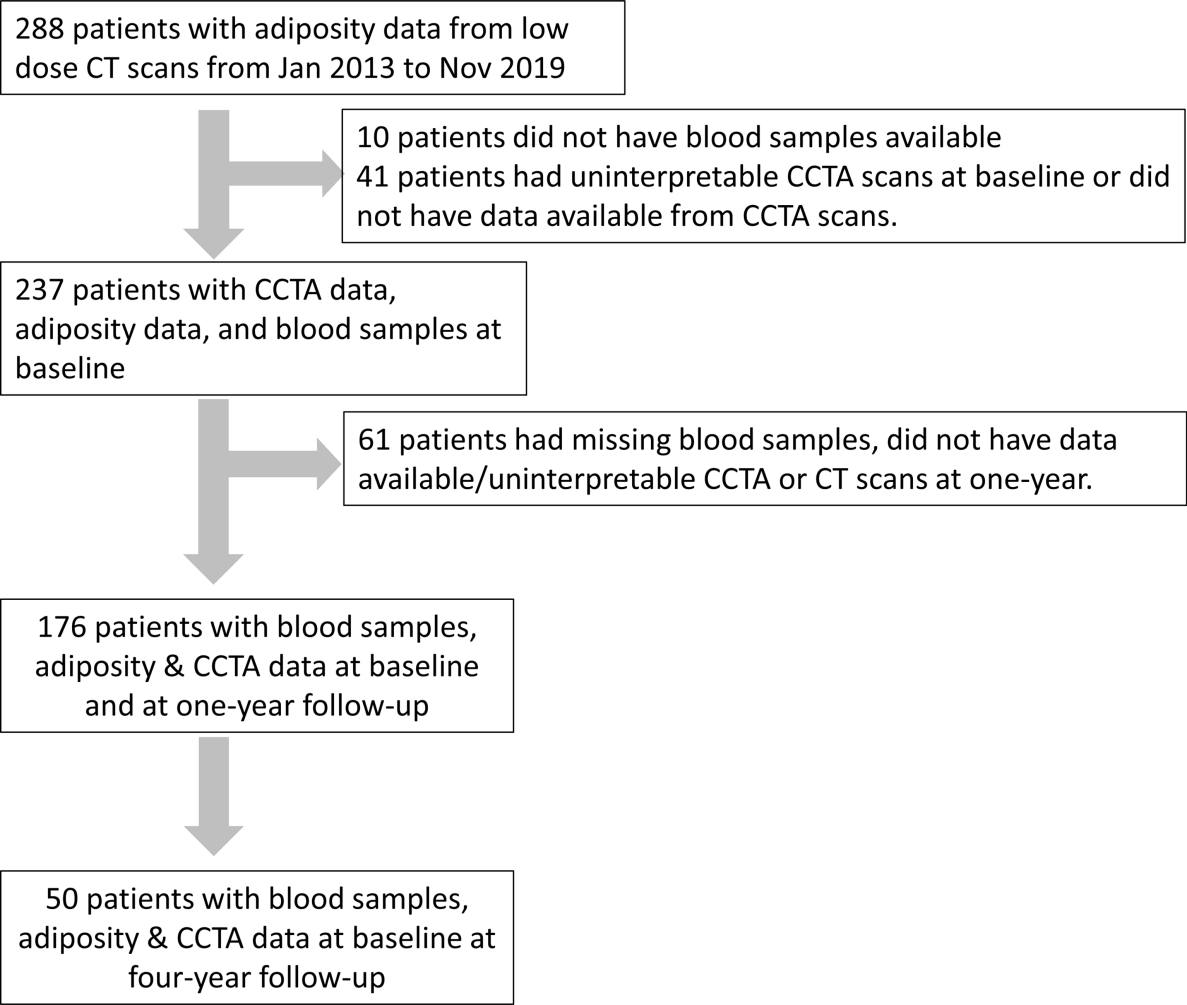
Recruitment and follow-up scheme. A total of 288 patients with psoriasis were examined at baseline. Of those, 237 consecutive psoriasis patients had quantitative data from CCTA scans at baseline. 176 patients were followed at 1 year and 50 were followed at 4 years.

A total of 288 patients with psoriasis were examined at baseline. They were recruited from our ongoing prospective observational cohort study between January 1, 2013, and November 1, 2019 (The Psoriasis Atherosclerosis Cardiometabolic Initiative) (Figure 5). Of those, 237 consecutive psoriasis patients had quantitative data from CCTA scans at baseline. 176 patients were followed at 1 year and 50 were followed at 4 years. The recruitment scheme for this study is summarized in Figure 5.

### Inclusion/exclusion criteria.

Patients with psoriasis were >18 years of age, required to have a formal diagnosis of plaque psoriasis by a dermatologist, and were examined by a certified health care provider to confirm the onset, duration, and severity of skin disease, as assessed by the PASI score. They underwent blood testing and CCTA imaging at baseline and 1-year and 4-year follow-ups. Patients with psoriasis were excluded from the study if they had estimated glomerular filtration rate <30 mL/min/1.73 m^2^; existing CV disease; any comorbid condition known to augment systemic inflammation, such as uncontrolled hypertension, internal malignancy within 5 years, human immunodeficiency virus, active infection within the past 72 hours of baseline, major surgery within the past 3 months, and pregnancy or lactation.

### Clinical data and laboratory measurements.

Clinical parameters included blood pressure, height, weight, and waist and hip circumferences. Skin disease severity was assessed using the PASI score. Laboratory parameters, including fasting blood glucose, fasting lipid panel, complete blood count, and systemic inflammatory markers (hs-CRP and GlycA) were measured at baseline and over time (at 1 and 4 years). Lipoprotein particle concentration and diameters were measured by NMR spectroscopy using methods previously described (LipoScience Inc.) (40). Systemic-biologic treatment included the following drugs: steroids, methotrexate, anti-TNF, anti–IL-12/IL-23, and anti–IL-17. A majority of the cohort underwent more psoriasis treatment at 1 year, and the same variables were recorded again after treatment.

### CCTA image acquisition and analysis.

Patients underwent CCTA using the same CT scanner (320-detector row Aquilion ONE ViSION, Toshiba). Scans were performed with prospective ECG gating at 100 or 120 kV tube potential, tube current of 100 to –850 mA adjusted to patient’s body size, and a gantry rotation time of 275 milliseconds. Image acquisition characteristics included slice thickness of 0.5 mm with slice increment of 0.25 mm. Blinded experienced readers evaluated coronary artery characteristics using semiautomated dedicated software (QAngio CT, Medis) as has been previously described (5). Coronary artery path lines were automatically extracted for each of the major epicardial coronary arteries >2 mm in diameter. Automated contouring of the inner lumen and outside wall was performed for each of the coronary arteries. Results were manually adjusted when clear deviations were present, and contours were reviewed on axial cross-sections at 0.5 mm increments for further adjustment. Coronary artery segmental plaque volume was indexed by length of the vessel to account for variable coronary artery lengths between patients and subsequently attenuated for luminal intensity to yield NCB and dense calcified burden using adaptive threshold for cutoff values (41). Test-retest reliability was analyzed using intraclass correlation coefficient (ICC) testing and demonstrated very good intraexaminer reliability (ICC = 95%).

### Measurement of adiposity.

VAT and SAT were quantified using low-dose CT. Abdominal adiposity was measured between the caudal end of the sternum and the cranial portion of the pubic symphysis, in which slices 50–150 were used in data analysis and averaged. An automated software was used with an active contour model algorithm on each designated CT slice. This algorithm and approach have been previously described (6, 42). A body mask was created in this algorithm, in which an anisotropic diffusion filter was used to reduce noise, and voxels between –274 and –49 Hounsfield were labeled as adipose tissue. An active contour model was used to create the internal contour, separating visceral and subcutaneous adipose tissues. Visceral adipose volume was then defined as the volume (cubic centimeters) of all adipose tissue voxels inside of the internal contour, and the rest of adipose volume was defined as subcutaneous adipose volume. Errors in configuration were screened and manually corrected by trained research fellows. Test-retest reliability was analyzed using ICC testing and demonstrated excellent intraexaminer reliability (ICC = 98%). Our primary analyses were meant to assess effects of visceral adipose volume on coronary disease in models adjusted for Framingham risk score (which includes sex), hs-CRP, statin therapy, biologic therapy, and SAT.

### Statistics.

Continuous data were reported as mean with standard deviation for parametric variables, median with IQR for nonparametric variables, and percentages for categorical variables. Student’s and paired *t* tests (2 tailed) were performed for parametric data, Mann-Whitney *U* and Kruskal-Wallis tests were used for nonparametric data, and Pearson’s χ^2^ test was performed for categorical variables. In baseline analyses, parametric and nonparametric variables were compared between the 2 groups using Student’s *t* test (2 tailed) and Mann-Whitney *U* test, respectively. For longitudinal analyses, patients were placed into 2 groups depending on whether they had increased or decreased VAT at 1 year.

Multivariable linear regression analyses were performed to evaluate the association between change in visceral adiposity and change in noncalcified coronary burden at 1 and 4 years, with adjustment for Framingham 10-year risk score, change in hs-CRP, change in SAT, baseline biologic and statin treatments, and 1-year biologic and statin treatments. If a patient started on statin treatment between the 2 time points, this would be reflected in the 1-year adjustment. Standardized β coefficient values were reported for these analyses, with *P* < 0.05 considered significant. Statistical analyses were performed using STATA 15 (Stata Corp.).

### Study approval.

The study protocols were approved by the institutional review board at the NIH. All study protocols are in compliance with the Declaration of Helsinki. All participants provided written informed consent after a full explanation of the procedures. STROBE guidelines were followed for this observational cohort study.

## Author contributions

AS and KMA contributed equally in study design, analysis, and manuscript writing. AS designed the study, acquired data, analyzed data, drafted the manuscript, and critically revised the manuscript. KMA designed the study, acquired data, analyzed data, drafted the manuscript, and critically revised the manuscript. ASR acquired data, analyzed data, drafted the manuscript, and critically revised the manuscript. AKD acquired data, analyzed data, drafted the manuscript, and critically revised the manuscript. DEU acquired data, analyzed data, and critically revised the manuscript. SSL analyzed data and critically revised the manuscript. AVS critically revised the manuscript. HLT acquired data and critically revised the manuscript. JC acquired data and critically revised the manuscript. JR acquired data and critically revised the manuscript. AAJ acquired data and critically revised the manuscript. YAE acquired data and critically revised the manuscript. AG acquired data and critically revised the manuscript. JAR acquired data and critically revised the manuscript. AK acquired data and critically revised the manuscript. JEA critically revised the manuscript. BL critically revised the manuscript. RP critically revised the manuscript. ES critically revised the manuscript. MPP acquired data, analyzed data, and critically revised the manuscript. MYC critically revised the manuscript. DAB critically revised the manuscript. JMG analyzed data and critically revised the manuscript. NNM designed the study, analyzed data, drafted the manuscript, critically revised the manuscript, supervised all parts of the study, and acquired funding for the study

## Supplementary Material

supplemental data

ICMJE disclosure forms

## Figures and Tables

**Table 1:**
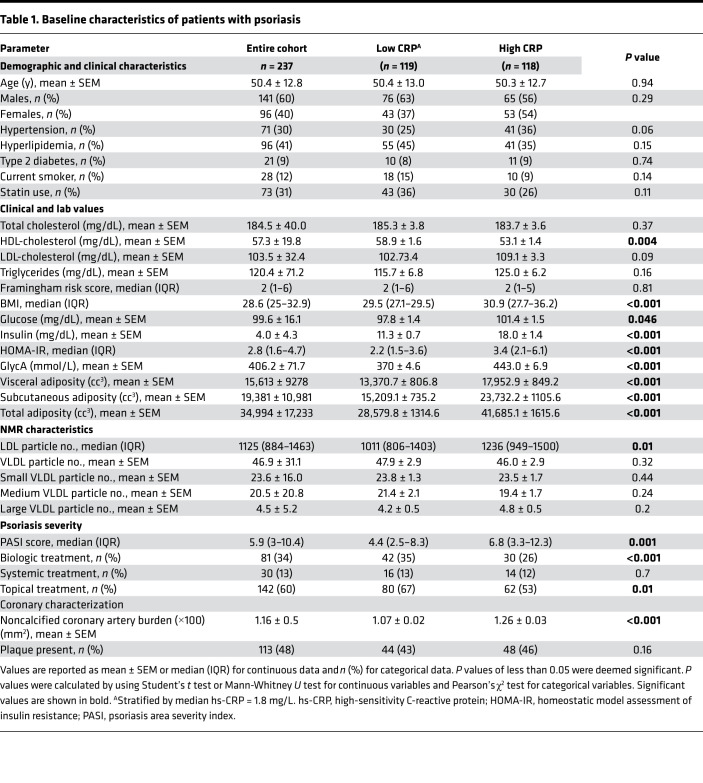
Baseline characteristics of patients with psoriasis

**Table 2:**
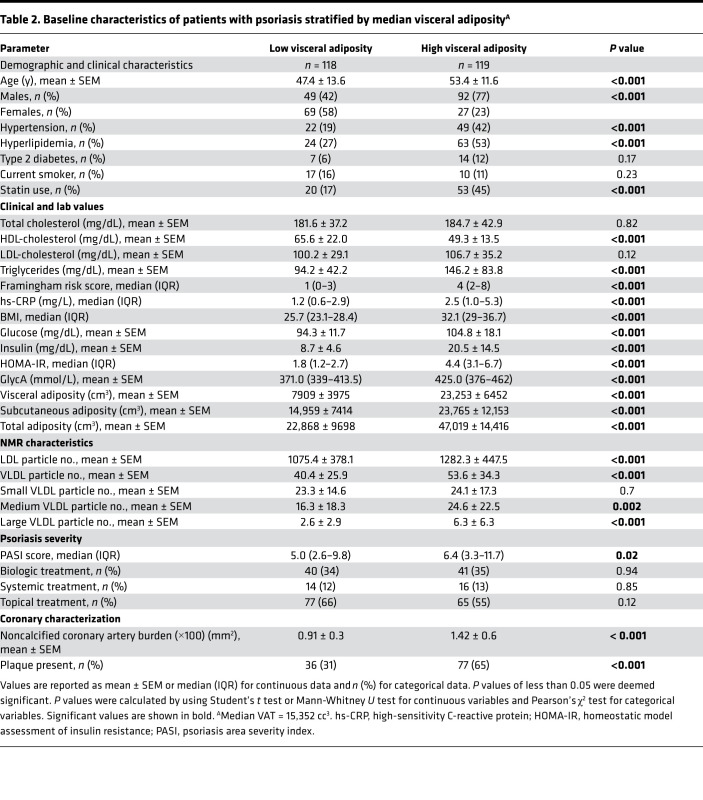
Baseline characteristics of patients with psoriasis stratified by median visceral adiposity^A^

**Table 3:**
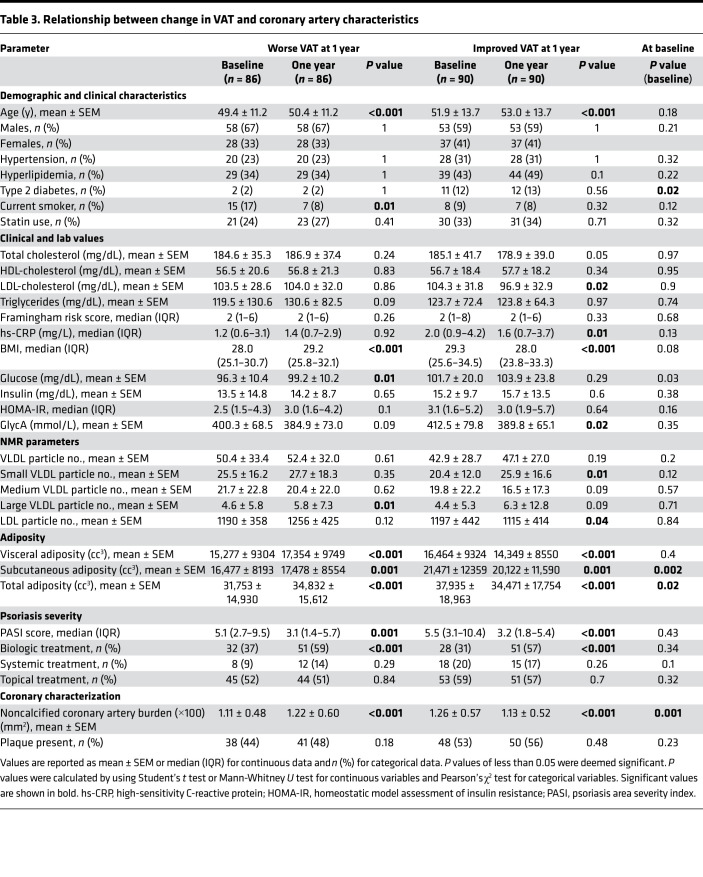
Relationship between change in VAT and coronary artery characteristics

## Version 1 10/26/2020

In-Press Preview

## Version 2 11/19/2020

Electronic publication
