# Supplementary material for: Chronic inflammation in psoriasis promotes visceral adiposity associated with noncalcified coronary burden over time
Source: JCI Insight. 2020 Nov 19;5(22):e142534. doi: 10.1172/jci.insight.142534 (PMC7710282; doi:10.1172/jci.insight.142534)
Supplement: supplemental data [file jciinsight-5-142534-s192.pdf]

Supplemental Table 1. Determinants of visceral adiposity

**Variable**	**Visceral Adiposity**	
**Demographics and clinical characteristics**	**Rho**	**P-value**
Age, years	0.31	**<0.001**
Males	0.42	**<0.001**
Hypertension	0.30	**<0.001**
Hyperlipidemia	0.28	**<0.001**
Diabetes mellitus	0.19	**<0.001**
Lipid therapy	0.38	**<0.001**
Current smoker	-0.08	**<0.001**
Framingham risk score	0.55	**<0.001**
Body mass index	0.71	**<0.001**
Waist to hip ratio	0.54	**<0.001**
**Clinical and lab values**		
Systolic blood pressure, mm Hg	0.20	**0.001**
Diastolic blood pressure, mm Hg	0.17	**0.004**
Total cholesterol, mg/dL	-0.05	0.43
HDL cholesterol, mg/dL	-0.49	**<0.001**
LDL cholesterol, mg/dL	0.07	0.25
Triglycerides, mg/dL	0.46	**<0.001**
Cholesterol efflux capacity	-0.32	**<0.001**
hs-CRP, mg/L	0.31	**<0.001**
Fasting blood glucose, mg/dL	0.47	**<0.001**
Insulin	0.67	**<0.001**
HOMA-IR	0.66	**<0.001**
Erythrocyte sedimentation rate, mm/hr	0.15	**0.01**
GlycA, μmol/L	0.37	**<0.001**
Subcutaneous adiposity	0.50	**<0.001**
**Psoriasis characterization**		
Psoriasis area severity index score	0.17	**0.004**
Biologic therapy	-0.01	0.85
**Coronary artery characterization**		
Prevalent plaque	0.40	**<0.001**
Non-calcified coronary artery burden, mm^2	0.55	**<0.001**
**NMR Parameters**		
LDL particle number	0.24	**<0.001**
Small LDL particle number	0.46	**<0.001**
Large LDL particle number	-0.24	**<0.001**
VLDL particle number	0.27	**<0.001**
Small VLDL particle number	0.004	0.95
Medium VLDL particle number	0.30	**<0.001**
Large VLDL particle number	0.51	**<0.001**

*****HDL indicates high-density lipoprotein; LDL, low-density lipoprotein; hs-CRP, high sensitivity-C reactive protein; HOMA-IR, homeostatic model assessment of insulin resistance; VLDL, very low-density lipoprotein; and PASI, psoriasis area severity index.

Supplemental Table 2. Patient characteristics at baseline and one year

**Parameter**	**Baseline**	**One-year**	**P-value**
**Demographics and medical history**	n=176	n=176	
Age, years	50.6 ± 12.5	51.7 ± 12.6	**<0.001**
Males, n (%)	111 (63)	111 (63)	1.00
Hypertension, N (%)	48 (27)	48 (27)	**0.03**
Hyperlipidemia, N (%)	68 (39)	73 (41)	0.34
Type 2 diabetes mellitus, N (%)	13 (7)	14 (8)	0.66
Current tobacco use, N (%)	23 (13)	14 (8)	**0.01**
Lipid treatment, N (%)	51 (30)	54 (31)	0.41
Framingham risk score	2.3 (0.7-6.1)	2.3 (1.0-6.0)	0.92
Body mass index	28.8 (25.2-32.9)	28.4 (24.6-32.3)	0.52
**Clinical and laboratory values**			
Total cholesterol, mg/dL	184.9 ± 38.6	182.8 ± 38.3	0.20
HDL cholesterol, mg/dL	56.6 ± 19.4	57.3 ± 19.7	0.21
LDL cholesterol, mg/dL	103.9 ± 30.2	100.3 ± 32.6	0.05
Triglycerides, mg/dL	121.6 ± 74.5	127.2 ± 73.6	0.15
GlycA, μmol/L	399 (355-444)	382 (337-426)	**0.003**
hs-CRP, mg/L	1.8 (0.8-3.8)	1.5 (0.7-3.6)	0.07
**Psoriasis characterization**			
Psoriasis area severity index score	5.4 (3.0-10.0)	3.2 (1.5-5.5)	**<0.001**
Biologic therapy, N (%)	60 (34)	102 (58)	**<0.001**
**Adipose characterization**			
Visceral adiposity (cc^3)	15883.9 ± 9306.9	15817 ± 9253.0	0.38
Subcutaneous adiposity (cc^3)	19030.5 ± 10796.4	18830.0 ± 10276.8	0.21
**Coronary Artery Characterization**			
Total coronary burden, mm^2	1.25 ± 0.55	1.24 ± 0.56	0.29
Non-calcified coronary artery burden, mm^2	1.19 ± 0.53	1.17 ± 0.56	0.27
Plaque Present (%)	86 (49)	91 (52)	0.17
**NMR Parameters**			
HDL particle number	33.8 (30.7-39.5)	34.7 (31.0-39.1)	0.27
Small HDL particle number	19.8 (14.7-23.6)	20.0 (14.5-24.5)	0.78
Medium HDL particle number	7.6 (4.6-12.4)	8.1 (3.9-13.1)	0.34
Large HDL particle number	5.4 (3.0-8.4)	5.6 (3.3-8.7)	0.06
LDL particle number	1156 (897-1484)	1165 (909-1449)	0.73
Small LDL particle number	455 (311-684)	479 (278-696)	0.68
Large LDL particle number	339 (203-523)	403 (216-536)	0.36
VLDL particle number	41.8 (24.1-62.3)	45.6 (30.0-63.1)	0.19
Small VLDL particle number	20.7 (11.6-33.8)	23.8 (14.0-36.9)	**0.02**
Medium VLDL particle number	14.6 (6.8-25.6)	12.8 (5.4-24.9)	0.26
Large VLDL particle number	2.5 (1.2-5.7)	3.4 (2.0-6.5)	**<0.001**

* HDL indicates high-density lipoprotein; LDL, low-density lipoprotein; hs-CRP, high sensitivity-C reactive protein; HOMA-IR, homeostatic model assessment of insulin resistance; VLDL, very low-density lipoprotein; and PASI, psoriasis area severity index.

Supplemental Table 3. Patient characteristics at baseline and four-years

Parameter	Baseline	Four-year	P-value
**Demographic and Clinical Characteristics**	n=50	n=50	
Age, years	51.9 ± 12.0	56.2 ± 11.9	**<0.001**
Males	29 (58)	29 (58)	1.00
Hypertension	17 (34)	12 (24)	0.10
Hyperlipidemia	28 (56)	21 (42)	**0.03**
Type-2 diabetes	5 (10)	3 (6)	0.32
Current smoker	6 (12)	7 (14)	0.65
Statin use	17 (34)	20 (40)	0.18
**Clinical and Lab Values**			
Total cholesterol, mg/dL	181.1 ± 43.4	181.0 ± 44.2	0.99
HDL cholesterol, mg/dL	53.6 ± 20.4	54.9 ± 18.5	0.42
LDL cholesterol, mg/dL	102.3 ± 32.6	97.8 ± 35.2	0.38
Triglycerides, mg/dL	130.8 ± 99.1	144.4 ± 92.3	0.44
Framingham risk score	4 (1-7)	4 (1-8)	0.57
hs-CRP, mg/L	1.8 (1.0-4.2)	2.1 (0.6-4.8)	0.78
Body Mass Index (BMI)	29.0 (26.1-35.7)	29.6 (25.8-33.0)	0.48
Glucose, mg/dL	99.0 ± 16.9	106.1 ± 33.8	0.04
Insulin, mg/dL	15.1 ± 8.9	15.7 ± 13.5	0.71
HOMA-IR	3.3 (1.7-4.6)	2.9 (1.7-5.4)	0.87
Visceral Adiposity (cm^3)	17986 ± 8978	18188 ± 8298	0.37
Subcutaneous Adiposity (cm^3)	21304 ± 12345	20937 ± 11040	0.26
Total Adiposity (cm^3)	39290 ± 18376	39125 ± 16012	0.44
**Psoriasis Severity**			
PASI Score	4.0 (1.8-7.7)	1.6 (0.9-3.6)	**0.003**
Biologic treatment	23 (46)	29 (58)	**0.004**
**Coronary Characterization**			
Non-calcified coronary artery burden, mm^2	1.24 ± 0.56	1.25 ± 0.49	0.37
Plaque Present (%)	28 (56)	29 (58)	0.56

***** HDL indicates high-density lipoprotein; LDL, low-density lipoprotein; hs-CRP, high sensitivity-C reactive protein; HOMA-IR, homeostatic model assessment of insulin resistance; VLDL, very low-density lipoprotein; and PASI, psoriasis area severity index.
